# Possible observation of highly itinerant quantum magnetic monopoles in the frustrated pyrochlore Yb_2_Ti_2_O_7_

**DOI:** 10.1038/ncomms10807

**Published:** 2016-02-25

**Authors:** Y. Tokiwa, T. Yamashita, M. Udagawa, S. Kittaka, T Sakakibara, D. Terazawa, Y. Shimoyama, T. Terashima, Y. Yasui, T. Shibauchi, Y. Matsuda

**Affiliations:** 1 Department of Physics, Kyoto University, Kyoto 606-8502, Japan; 2 Research Center for Low Temperature and Materials Science, Kyoto University, Kyoto 606-8501, Japan; 3 Department of Physics, Gakushuin University, Mejiro, Toshima-ku, Tokyo 171-8588, Japan; 4 Institute for Solid State Physics, University of Tokyo, Kashiwa 277-8581, Japan; 5 Department of Physics, School of Science and Technology, Meiji University, Higashi-mita, Tama-ku, Kawasaki 214-8571, Japan; 6 Department of Advanced Materials Science, University of Tokyo, Kashiwa 277-8561, Japan

## Abstract

The low-energy elementary excitations in frustrated quantum magnets have fascinated researchers for decades. In frustrated Ising magnets on a pyrochlore lattice possessing macroscopically degenerate spin-ice ground states, the excitations have been discussed in terms of classical magnetic monopoles, which do not contain quantum fluctuations. Here we report unusual behaviours of magneto-thermal conductivity in the disordered spin-liquid regime of pyrochlore Yb_2_Ti_2_O_7_, which hosts frustrated spin-ice correlations with large quantum fluctuations owing to pseudospin-1/2 of Yb ions. The analysis of the temperature and magnetic field dependencies shows the presence of gapped elementary excitations. We find that the gap energy is largely suppressed from that expected in classical monopoles. Moreover, these excitations propagate a long distance without being scattered, in contrast to the diffusive nature of classical monopoles. These results suggests the emergence of highly itinerant quantum magnetic monopole, which is a heavy quasiparticle that propagates coherently in three-dimensional spin liquids.

## Abstract (web summary)

Spin excitations above the ground state of spin ices, geometrically frustrated pyrochlore magnets, are equivalent to pairs of oppositely charged deconfined magnetic monopoles which may propagate through the system. Here, the authors evidence highly itinerant quantum magnetic monopole excitations in Yb_2_Ti_2_O_7_.

## Graphical abstract

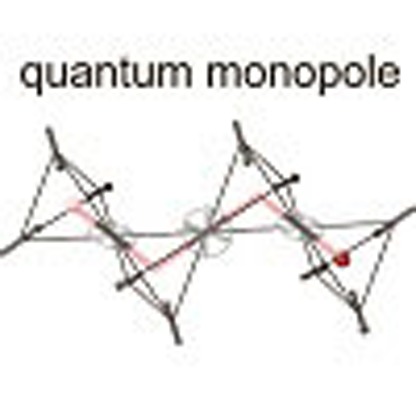

Rare-earth pyrochlore oxides exhibit various exotic magnetic properties owing to their strong geometrical frustration experienced by coupled magnetic moments on the tetrahedral lattice (Fig. 1a)^1^. The most explored materials are Ho_2_Ti_2_O_7_ and Dy_2_Ti_2_O_7_, in which the magnetic moments can be regarded as classical spins with a strong easy-axis (Ising) anisotropy^1^,^2^. The frustration of these moments results in a remarkable spin ice with macroscopically degenerate ground states, in which each tetrahedron has the two spins in and two spins out (2-in-2-out) configuration. This spin structure is characterized by dipolar spin correlations with a power law decay, which is observable as the unusual pinch-point shape of spin structure factor by neutron scattering^3^,^4^. One of the most remarkable features of the spin-ice state is that it hosts emergent magnetic monopole excitations; the first excitation is a 3-in-1-out configuration^5^,^6^. This produces a bound pair of north and south poles, which can be fractionalized into two free magnetic monopoles. This classical monopole excitations are gapped and dispersionless (Fig. 1b). Therefore the propagation of monopoles occurs only diffusively and the monopole population decays exponentially at temperatures well below the gap. Of particular interest is how the spin-ice ground state is altered by the quantum fluctuations, which may lift the degeneracy of the spin-ice manifold, leading to a new ground state such as quantum spin-ice state^7^,^8^,^9^,^10^,^11^. To clarify this issue, uncovering newly emergent elementary excitations in the presence of quantum fluctuations is crucially important. Although exotic excitations such as gapless photon-like mode have been proposed theoretically, the nature of excitations are poorly explored.

Among the magnetic pyrochlore materials, Yb_2_Ti_2_O_7_, Er_2_Ti_2_O_7_, Pr_2_Sn_2_O_7_ and possibly Tb_2_Ti_2_O_7_ host strong quantum fluctuations of magnetic dipoles owing to pseudospin-1/2 of magnetic rare-earth elements^12^,^13^,^14^,^15^. In particular, Yb_2_Ti_2_O_7_ is a good model system to study the influence of the quantum effects on monopole excitations. This is because the low-temperature physical properties are not influenced by the crystalline electric field-excited levels owing to the well separated excited levels from the ground state^16^. In addition, the full set of Hamiltonian parameters has been determined by inelastic neutron scattering experiments^12^,^16^. The Hamiltonian consists of three main interactions, *J*_||_, *J*_⊥_ and *J*_*z*±_. Here *J*_||_ (=2 K) is the Ising component of the nearest neighbour interaction, *J*_⊥_(=0.58 K) is the *XY*-component and *J*_*z*±_(=1.7 K) is the off-diagonal component. Finite *J*_⊥_ and *J*_*z*±_ produce quantum fluctuations (Fig. 1c). In Yb_2_Ti_2_O_7_, *J*_*z*±_, which is comparable to *J*_||_, gives rise to dispersive monopole excitations, that is, itinerant magnetic monopoles. Yb_2_Ti_2_O_7_ undergoes a first-order ferromagnetic phase transition at *T*_C_∼0.2 K (refs 17, 18). It is widely believed that quantum fluctuations keep spins from freezing and lead to a spin-liquid state. Therefore, the pinch-point structure observed in the ferromagnetic samples by neutron scattering above *T*_C_ indicates spin-liquid phase with spin-ice correlations.

Here, to study the elementary excitations in the spin-liquid state of Yb_2_Ti_2_O_7_, we measured the thermal conductivity, which is a powerful probe for low-energy excitations at low temperatures, providing a sensitive measurement of a flow of entropy conducted by magnetic excitations and phonons. The thermal conductivity has been reported in the classical spin-ice state of Dy_2_Ti_2_O_7_ recently^19^,^20^. However, the interpretation of the thermal conductivity of Dy_2_Ti_2_O_7_ appears to be complicated owing to the strongly suppressed phonon thermal conductivity by unknown additional scatterings (Supplementary Fig. 1; Supplementary Note 1). In fact, suggested heat transport by classical monopole is at odds with the diffusive motion of the dispersionless classical monopoles. We show that the thermal conductivity of Yb_2_Ti_2_O_7_ is rather simple. The monopole thermal conductivity can be well separated from the phonon contribution, which obeys magnetic field/temperature (*H*/*T*) scaling. Our analysis shows the evidence of the substantial heat transport by quantum magnetic monoples, whose excitation energy is significantly suppressed from that of classical monopoles. The quantum magnetic monopoles become itinerant due to quantum fluctuations, in stark contrast to the localized and diffusive nature of classical monopoles.

## Results

### Thermal conductivity and specific heat at zero magnetic field

Figure 2a shows the temperature dependence of thermal conductivity divided by temperature *κ*/*T* in zero field and at *μ*_0_*H*=12 T measured on a single crystal of Yb_2_Ti_2_O_7_, where *μ*_0_ is the vacuum permeability. Distinct jump in *κ*/*T* at zero field is observed at *T*_C_. As shown in Fig. 2b, the specific heat *C* of the single crystal taken from the same batch shows a sharp and large jump at the same *T*_C_ (ref. 17). We note that there are also some experiments reporting the absence of long-range order even below the critical temperature^21^,^22^,^23^,^24^,^25^. However, in the previous studies, a sharp single jump in *C*/*T* had been reported only in the powdered samples, but not in single crystals^21^,^26^. In contrast, in the recent high-quality single crystals having a sharp *C*/*T* jump, the long-range ferromagnetic ordering below *T*_C_ and the pinch-point features in neutron scattering have been clearly observed^17^,^18^. As shown in Fig. 2c, zero field *κ*/*T* above *T*_C_ follows a *T*-linear dependence with negligibly small intercept at *T*=0 K. The absence of residual 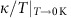 in the spin-liquid state with spin-ice correlations will be discussed later.

### Magneto-thermal conductivity

As clearly seen in Fig. 2a, magnetic field strongly enhances the thermal conductivity. Figure 3a–d show the field dependence of *κ*(*H*)/*T* for different field directions. As illustrated in the inset of Fig. 3e, there are three characteristic regimes; low-field regime where *κ*(*H*)/*T* decreases with *H*, intermediate-field regime where *κ*(*H*)/*T* increases, and high-field regime where *κ*(*H*)/*T* exhibits a saturation.

In the present system, heat is transferred by phonons and magnetic excitations: *κ*=*κ*_p_+*κ*_m_. We point out that the field dependence of *κ*(*H*)/*T* in the intermediate- and high-field regimes are dominated by the phonon contribution *κ*_*p*_ determined by spin-phonon scattering, which contains elastic and inelastic processes. The elastic scattering is enhanced with increasing disorder of spins and thus this scattering process should be monotonically suppressed by the alignment of spins with increasing magnetic field. A recent calculations of magnetoresistance in a fluctuating spin-ice state indicates that the electron-spin elastic scattering rate decreases with increasing magnetization^27^, which supports this trend. The inelastic scattering is directly related to the quantum dynamics of spin. In this inelastic scattering, the leading spin-flip process accompanies a hopping of a monopole to the neighbouring tetrahedron (which is related to *J*_⊥_), because this process requires much lower energies than creation or annihilation of monopoles. This scattering is suppressed with field by the formation of Zeeman gap. (see Supplementary Note 2 for discussion in more detail.) Therefore an external magnetic field suppresses both elastic and inelastic scatterings, leading to the enhancement of the phonon thermal conductivity *κ*_p_.

In the high-field regime, the Zeeman splitting energy *gμ*_B_*H* well exceeds both of the magnetic interactions and thermal energy, *gμ*_B_*H*
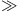
*J*_||_, *J*_⊥_, *J*_*z*±_ and *k*_B_*T*, where *g* is the *g*-factor, *μ*_B_ is the Bohr magneton and *k*_B_ is the Boltzmann constant. In this situation, where all spins are fully polarized and the magnetic (spin-wave) excitations are gapped with a gap *gμ*_B_*H*, thermal conductivity is almost entirely dominated by the pure phonon contribution without spin scattering because of the following reasons. First, elastic spin-phonon scattering is absent due to the perfect alignment of spins. Second, inelastic scattering is also absent due to the formation of the large Zeeman gap. Third, spins do not carry the heat due to the Zeeman gap. As purely phononic thermal conductivity is insensitive to magnetic field, *κ*(*H*)/*T* in the high-field regime is nearly independent of *H*. In the intermediate-field regime, the phonon mean free path is significantly reduced by the spin-phonon scattering due to the spins thermally excited across the Zeeman gap. In fact, as shown in Fig. 3e which plots *κ*/*T* as a function of *μ*_B_*H*/*k*_B_*T*, all data collapse into a single curve except for the low *μ*_B_*H*/*k*_B_*T* regime. The fact that data for both field directions stabilizing different spin configurations (3-in-1-out for *H* || [1, 1, 1] and 2-in-2-out for *H* || [0, 0, 1]) follow the same curve implies that the elastic spin-phonon scattering dominates over the inelastic scattering in this regime. It is intriguing that the *H*/*T* scaling curve appears to follow the Brillouin function (the dashed line in Fig. 3e). This coincidence with the Brillouin function calls for further theoretical investigations.

**Figure 1:**
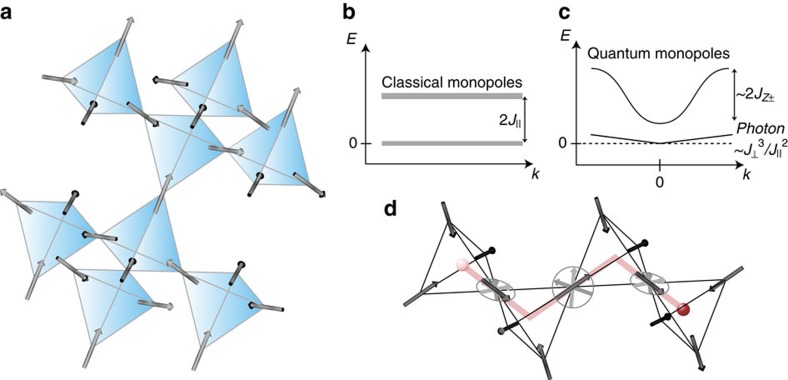
Monopole excitations in the 3D pyrochlore lattice. (**a**) Spin-ice structure on frustrated pyrochlore lattice. (**b**) Magnetic monopole excitation in classical spin ice. Monopole excitation energy, *E*=2*J*_||_, is dispersionless without dependence on wave number, *k*. (**c**) Quamtum magnetic monopole and photon excitations in quantum spin ice. Quantum monopole excitation becomes dispersive due to off-diagonal interaction *J*_*z*±_. The photon excitation based on the *XY*-component *J*_⊥_ is gapless and has liner dispersion. (**d**) Collective motion of quantum magnetic monopoles.

A particularly important information for the elementary excitations is provided by *κ*(*H*)/*T* in the low-field regime, where *κ*(*H*)/*T* decreases with *H* (Fig. 3c,d) and exhibits clear deviations from the *H*/*T* scaling curve (Fig. 3e). This low-field behaviour of *κ*(*H*)/*T* arises from the magnetic excitations because the initial reduction with *H* cannot be explained by spin-phonon scattering, which always increases *κ*(*H*) with *H* as discussed above. We point out that the magnetic monopoles are most likely to be responsible for this excitations because of the following reasons. First, the deviations in the low-field regime appear below a characteristic temperature T*∼4 K, where the pinch-point features in neutron scattering appears^17^. In addition, T* is close to the temperature 2*J*_||_/*k*_B_, above which monopole excitation disappears. Second, as shown in Fig. 3c (Supplementary Figs 2, 3 and 4; Supplementary Notes 3 and 4), the initial reduction of *κ*(*H*)/*T* disappears below *T*_C_, which is consistent with the monopole scenario because ferromagnetic ordering prevents the monopole formation. Third, the effect of ferromagnetic fluctuations as the origin of the anomalous low-field behaviour is safely excluded, as we discuss below. Since classical localized monopoles do not transport the heat, these results suggest the emergence of itinerant quantum magnetic monopoles illustrated in Fig. 1c,d.

The appearance of the quantum monopoles are supported by the field dependence of *κ*(*H*)/*T* shown in Fig. 3c,d. The decrease of *κ*(*H*)/*T* with *H* implies that the number of monopoles is reduced with *H* at low fields. This initial reduction is expected in the dispersionless classical monopoles with gap 2*J*_||_ (Supplementary Fig. 5; Supplementary Note 5). However, in the classical case, the number of monopoles will decay exponentially with decreasing temperature below T*∼2*J*_||_/*k*_B_. Therefore the observed quite substantial reduction of *κ*(*H*)/*T* even at low temperatures well below T* is inconsistent with the classical monopoles. The results indicate that the monopole excitation gap is largely suppressed from the classical monopole, which is consistent with the dispersive quantum monopoles (Fig. 1c). We also note that the substantial reduction of monopole density by the low field will result in a reduction of the inelastic spin-phonon scattering process related to the monopole hopping discussed above, which further emphasizes the significant role of the quantum monopoles themselves as a heat conducting carrier at low fields.

Here we comment on the field direction dependence of *κ*(*H*)/*T*. As shown in Fig. 3a,b,e, *κ*(*H*)/*T* is nearly isotropic with respect to the *H*-direction at high fields. Anisotropic *κ*(*H*)/*T* may be expected at high fields, because with increasing *H*, the monopole density increases for *H* || [1, 1, 1], while it decreases to zero for *H* || [0, 0, 1] (Supplementary Fig. 5; Supplementary Table 1). However, monopoles tend to localize at high fields because the energy of spin-flip, which is necessary for the monopole propagation, increases linearly with *H*. Thus monopole propagation does not contribute to the thermal conductivity at high fields, which is consistent with the observed isotropic *κ*(*H*)/*T*.

**Figure 2:**
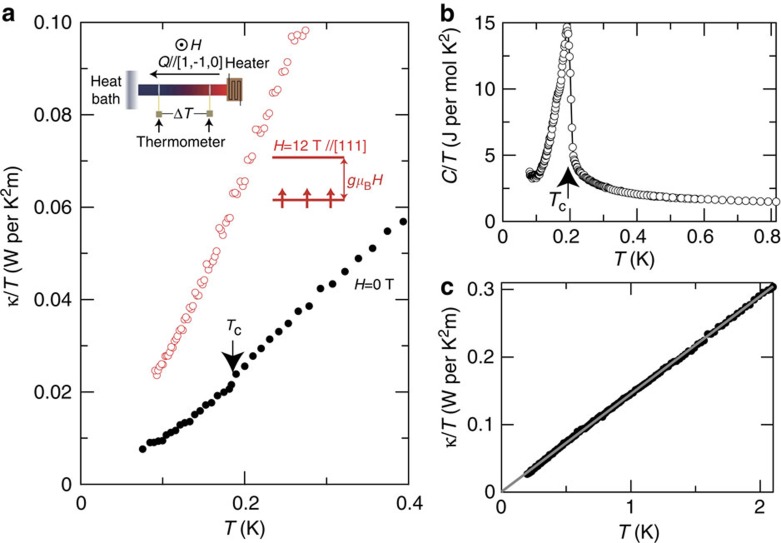
Low-temperature thermal conductivity and specific heat of Yb_2_Ti_2_O_7_. (**a**) *κ*/*T* at zero and *μ*_0_*H*=12 T applied along [1, 1, 1] direction is plotted against temperature. The heat current is applied along [1, −1, 0]. At *T*_C_, *κ*/*T* exhibits a jump, indicated by an arrow. Inset illustrates the measurement configuration of the thermal conductivity. (**b**) Specific heat divided by temperature *C*/*T* at zero field. (**c**) *κ*/*T* at zero field in the spin-liquid state above 0.2 K. Grey line is a fit to a *T*-linear dependence *κ*/*T*=*AT* with *A*=0.15 W per K^2^m.

As shown in the inset of Fig. 4, *κ*(*H*) decreases as *κ*(*H*)=*κ*(0)−*αH*^2^ (*α*>0) at very low fields. As the thermal conduction by magnetic excitations is determined by the number of low-energy itinerant quasiparticles, this *α* is a measure of the suppression rate of magnetic monopoles at low fields. Figure 4 depicts the temperature dependence of *α* for *H* || [0, 0, 1] and [1, 1, 1]. As the temperature is lowered, *α* first increases, decreases after showing a maximum at 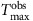
=0.3–0.5 K and suddenly vanishes at *T*_C_. Here, we stress that the initial reduction of *κ*(*H*) is not caused by ferromagnetic fluctuations, since monotonic increase of ferromagnetic fluctuations with approaching *T*_C_ is inconsistent with the non-monotonic temperature dependence of *α*. The difference in the magnitude of *α* in the two field directions may be related to the expected difference in the density of 3-in-1-out configuration at high fields, but the trends of *α*(*T*) are similar in both cases. The enhancement of *α* with decreasing *T* below T* can be accounted for by the reduction of thermal smearing of the monopole excitations. At low temperatures below the gap energy of monopole excitation, the monopole density decreases rapidly with decreasing temperature, leading to a suppression of *α*. As a result, *α* exhibits non-monotonic temperature dependence with a maximum. The calculation shows that the initial suppression of the monopole density reaches a maximum at around *T*_max_≈Δ/2.5*k*_B_, where Δ is the monopole excitation gap (Supplementary Fig. 6; Supplementary Note 5). In Yb_2_Ti_2_O_7_, assuming that monopole band minimum is lowered by ∼*J*_*z*±_ due to its quantum motion, Δ/*k*_B_=(2*J*_||_−*J*_*z*±_)/*k*_B_ is estimated to be ∼2 K, which yields *T*_max_∼0.8 K. The fact that 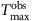 compares with *T*_max_ suggests that the maximum of *α* appears as a result of gap, which is largely suppressed from the classical one (2*J*_||_/*k*_B_∼4 K). We point out that the further reduction of 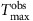 from *T*_max_ may be due to the influence of the quantum fluctuations on the velocity and mean free path included in the thermal conductivity. The present results lead us to conclude that the thermally excited quantum monopoles carry substantial portion of the heat particularly in the low-field regime. This is reinforced by the fact that *κ*/*T* at zero field shows a distinct decrease below *T*_C_ (Fig. 2a), where the phonon contribution *κ*_p_ is expected to be enhanced owing to the ferromagnetic spin alignment.

### Estimation of mean free path

**Figure 3:**
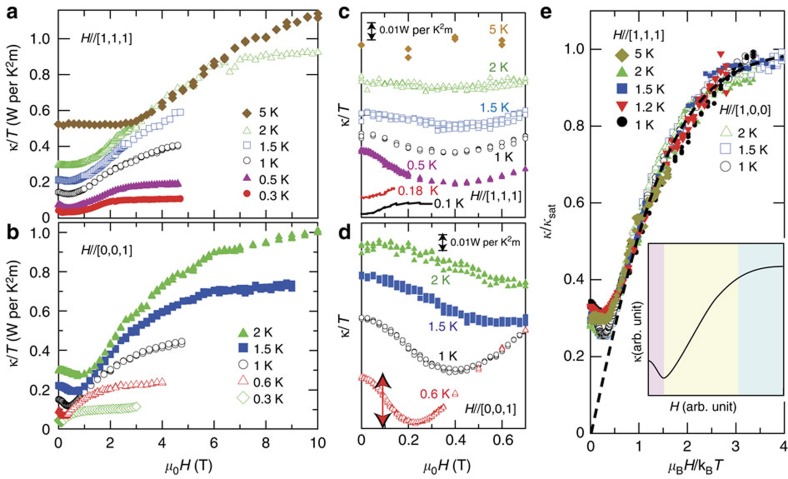
Heat conduction under magnetic fields. (**a**,**b**) Field dependence of *κ*/*T* of Yb_2_Ti_2_O_7_ for *H* || [1, 1, 1] and [0, 0, 1] with the heat current along [1, −1, 0]. (**c**,**d**) Field dependence of *κ*/*T* at low field. Data are shifted vertically for clarity. Double-headed red arrow indicates the initial reduction of *κ*(*H*)/*T* for *H* || [0, 0, 1] at *T*=0.6 K, which is estimated to be 0.03 W per K^2^m, giving a lower-bound estimate of the monopole contribution. (**e**) Normalized thermal conductivity *κ*/*κ*_sat_ plotted against *μ*_B_*H*/*k*_B_*T*, where *κ*_sat_ is the saturated thermal conductivity at high fields. *κ*_sat_ at high temperatures is determined so as to fit the scaling curve. The dashed line represents the Brillouin function with spin=1/2, assuming *g*=0.79. The inset illustrates the typical behaviour of *κ*(*H*)/*T*. There are three characteristic regimes, low-, intermediate- and high-field regimes, which are indicated by pink, yellow and blue colours, respectively.

**Figure 4:**
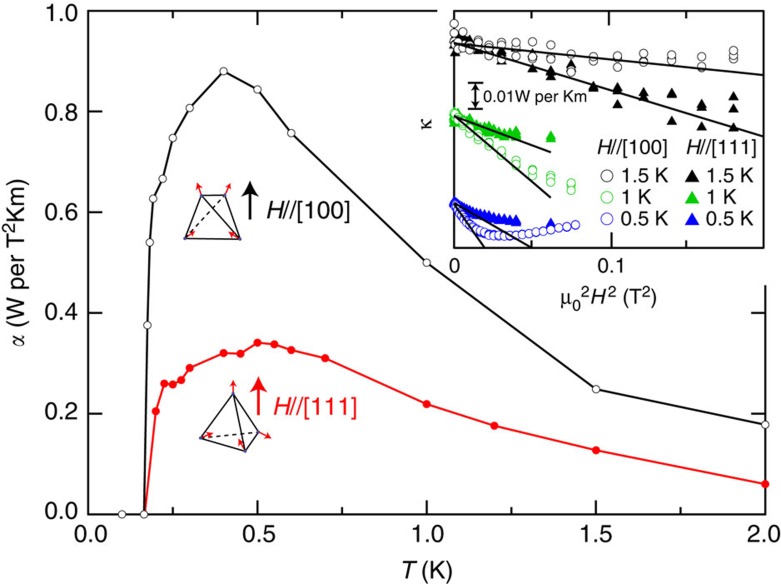
*H*^2^ reduction rate of monopole thermal conductivity as a function of temperature. Temperature dependence of the initial slope of *κ*(*H*) determined by fitting *κ*(*H*)=*κ*(0)−*αH*^2^, for *H* || [0, 0, 1] and [1, 1, 1]. Inset shows *κ*(*H*) plotted as a function of *H*^2^ at very low field.

Next we demonstrate that quantum monopoles are highly itinerant in the crystal lattice. Assuming the kinetic approximation, the monopole contribution to the thermal conductivity *κ*_m_ is written as 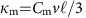, where *C*_m_ is the monopole contribution in the specific heat, *v* is the velocity and 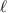 is the mean free path of the monopoles. We estimate 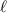 at 0.6 K simply by assuming that the amount of initial reduction of *κ*(*H*)/*T* shown by red double-headed arrow in Fig. 3d is attributed to the monopole contribution. The total specific heat *C*≈1 J/Yb-mol K at 0.6 K (Fig. 2b) and *v*, which is roughly determined by *v*∼*aJ*_*z*±_/2*πħ*∼15 m s^−1^, where *a*(=0.43 nm) is the distance between neighbouring tetrahedra, yield 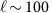 nm, or equivalently the scattering time 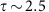 ns. We stress that this long 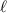 is still underestimated, since the total specific heat and the initial reduction of thermal conductivity give only an overestimate and underestimate, respectively, for the monopole contribution. This indicates that the excitations are mobile to a very long distance, 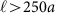, without being scattered. We note that 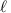 is much longer than the inter-monopole distance, which is estimated to be at most 5*a*, assuming monopole density of 1% of total number of tetrahedra. This corresponds to a very large coherent volume including more than ∼10^7^ tetrahedra, demonstrating highly itinerant transport of this long-lived particle, whose effective mass is as heavy as ∼2,000 times the bare electron mass^28^. This very small scattering rate may be due to the quantum feature, which prohibits the simple monopole–antimonopole pair annihilation that violates energy conservation.

## Discussion

The present results indicate the significant heat conduction by magnetic excitations, which are most likely magnetic monopoles. This implies that the monopole excitation becomes dispersive due to the off-diagonal term *J*_*z*±_ (Fig. 1c). This is consistent with the strongly suppressed monopole excitation gap, indicated by our analysis. We note that the observed nearly gapless excitations are not relevant to the photon excitations predicted by refs 7, 8, 9, 10, 11. This is because the characteristic photon energy 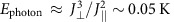 is one order of magnitude smaller than the present temperature range, and hence the strongly temperature dependent *α* is incompatible with the photon excitations.

The itinerant heavy quantum monopoles in the spin-liquid state appear to be a characteristic feature of the elementary excitations in frustrated magnetic pyrochlore systems with strong quantum fluctuations. Nearly ballistic propagation phenomena of fractionalized magnetic excitations in spin-liquid states have been reported in spin-1/2 one-dimensional Heisenberg chain^29^,^30^ and two-dimensional (2D) triangular lattice with antiferromagnetic interactions^31^. In the former elementary excitation is spinon which obeys semion statistics^32^ and in the latter excitation has been discussed in terms of spinon which obeys fermionic statistics^33^,^34^,^35^,^36^,^37^,^38^. In the present three-dimensional (3D) system elementary excitation in the spin-liquid state is quantum monopole, which is another fractionalized spinon. The residual 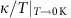, which is distinctly present in the 2D case^31^,^38^, is absent in Yb_2_Ti_2_O_7_ (Fig. 2c), implying that this 3D spinon is unlikely to be fermionic. In fact, bosonic spinon has been presumed theoretically in 3D pyrochlore lattice^39^,^40^. In one-dimensional Heisenberg system, the mean free path is infinite at nonzero temperature due to the integrability of the Hamiltonian. The highly itinerant fermionic spinons in 2D and bosonic quantum monopoles in 3D may be a key feature of the elementary excitations in highly frustrated quantum magnets and its origin is an open question.

## Methods

### Single-crystal growth

High-quality single crystals of Yb_2_Ti_2_O_7_ were grown by the floating zone method. Stoichiometric amount of Yb_2_O_3_ and TiO_2_ powder were mixed, pressed into rods and sintered at 1,150 °C for 24 h. Single crystals were grown from the rods in air at a rate of 1.5 mm h^−1^. The crystal ingot has a typical diameter of ∼6 mm and a length of ∼20 mm. Powder X-ray diffraction experiments on pulverized single crystal show no appreciable amount of impurity phase.

### Thermal conductivity and specific heat measurements

Thermal conductivity was measured along [1, −1, 0] direction by the standard steady-state method in a dilution refrigerator. Magnetic field was applied along [1, 1, 1] and [0, 0, 1], perpendicular to the heat current. As shown in the inset of Fig. 2a, the temperature difference within the sample, Δ*T*, due to the heat current from the heater to heat bath was measured by two Ruthenium oxide thermometers. Sample temperature was measured with high accuracy with use of alternating current resistance bridges. About 1 kΩ chip resistor was used for a heater. A single crystalline sample was well thermally coupled to the thermometers, heater and heat bath by thermally connecting with 50 μm silver wires and silver paint as a glue. Specific heat was determined by the standard quasi-adiabatic heat pulse method in a dilution refrigerator. Sample temperature was measured by ruthenium oxide thermometer and heat pulse was produced by Joule heating of resistive strain gauge attached to the sample.

## Additional information

**How to cite this article:** Tokiwa, Y. *et al*. Possible observation of highly itinerant quantum magnetic monopoles in the frustrated pyrochlore Yb_2_Ti_2_O_7_. *Nat. Commun.* 7:10807 doi: 10.1038/ncomms10807 (2016).

## Supplementary Material

Supplementary Information Supplementary Figures 1-6, Supplementary Table 1, Supplementary Notes 1-5 and Supplementary References
