# Supplementary material for: Possible observation of highly itinerant quantum magnetic monopoles in the frustrated pyrochlore Yb2Ti2O7
Source: Nat Commun. 2016 Feb 25;7:10807. doi: 10.1038/ncomms10807 (PMC4773418; doi:10.1038/ncomms10807)
Supplement: Supplementary Information — Supplementary Figures 1-6, Supplementary Table 1, Supplementary Notes 1-5 and Supplementary References [file ncomms10807-s1.pdf]

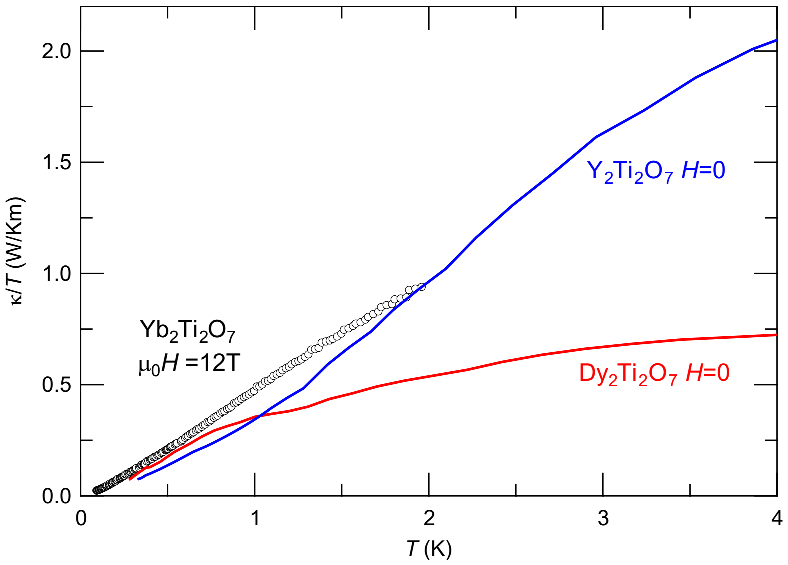


**Supplementary Figure 1.** $\kappa/T$ of $Y_2Ti_2O_7$, $Dy_2Ti_2O_7$ at zero field and $Yb_2Ti_2O_7$ at 12-T field applied parallel to [1,1,1] with the same heat current direction along [1,-1,0]. Data of $Y_2Ti_2O_7$, $Dy_2Ti_2O_7$ are taken from Ref. [1].

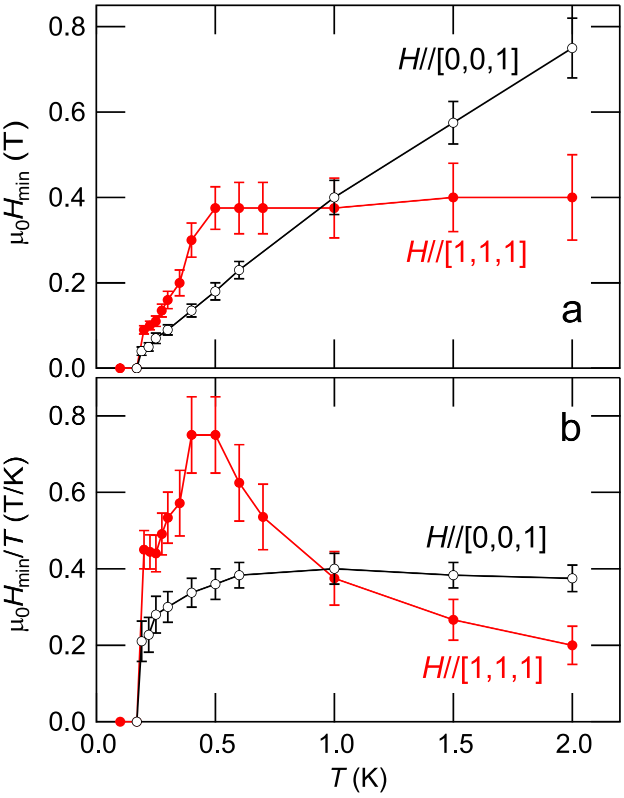


**Supplementary Figure 2.** **a**, Position of minimum in $\kappa(H)$, $\mu_0 H_{min}$, and **b** $\mu_0 H_{min}$ divided by temperature as a function of temperature. Error in $\mu_0 H_{min}$ is originated from the uncertainty in determination of $\mu_0 H_{min}$ due to scattering of data.

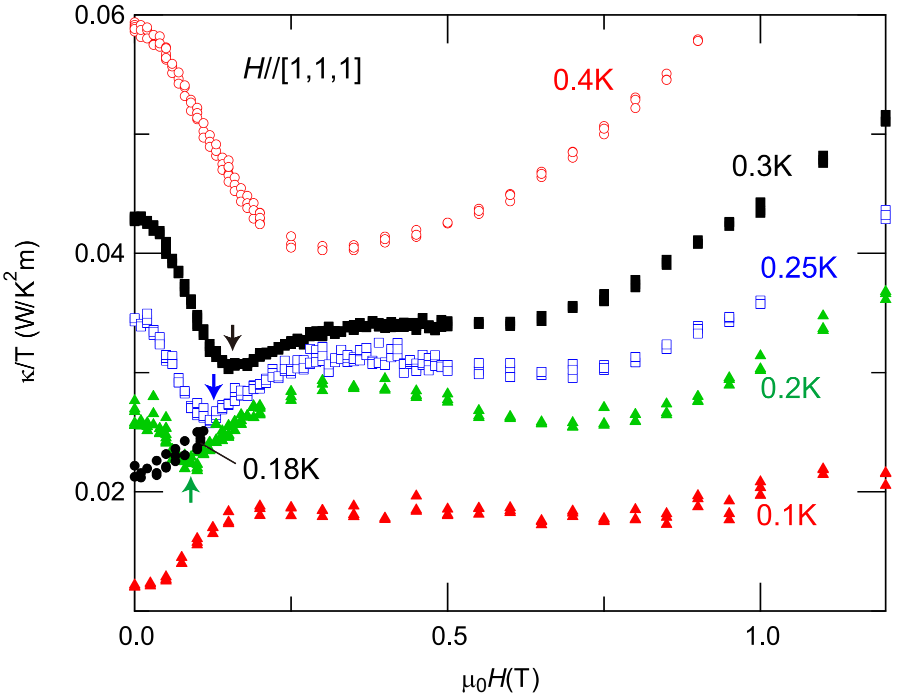


**Supplementary Figure 3.** Field dependence of $\kappa/T$ of $Yb_2Ti_2O_7$ for $\boldsymbol{H}$//[1,1,1] with the heat current along [1,-1,0]. Arrows indicate the field-induced transition from paramagnetic to ferromagnetic states.

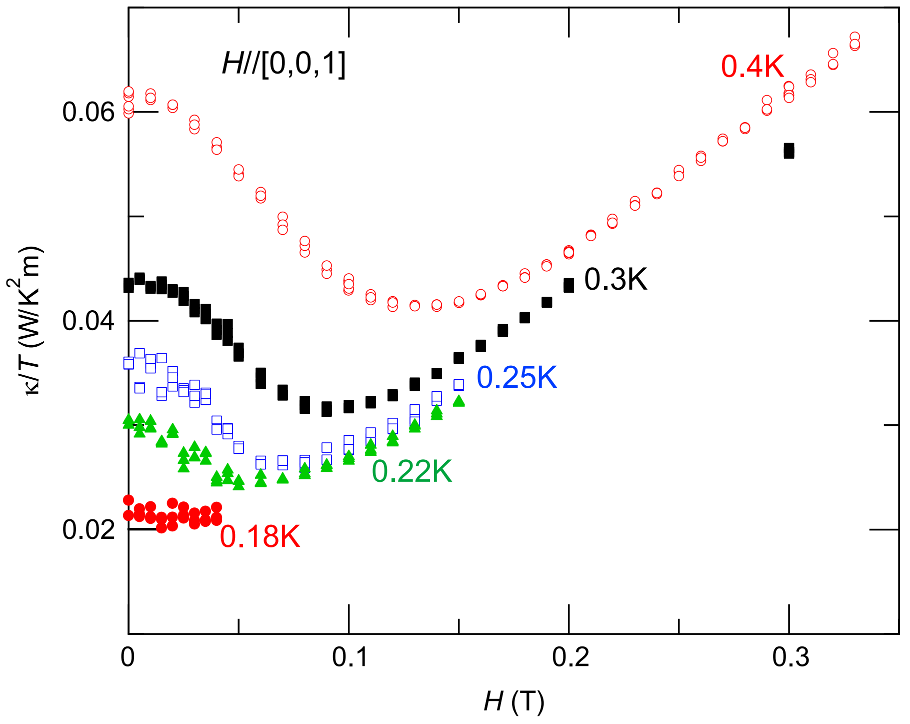


**Supplementary Figure 4.** Field dependence of $\kappa/T$ of $Yb_2Ti_2O_7$ for $\boldsymbol{H}$//[0,0,1] with the heat

current along [1,-1,0].

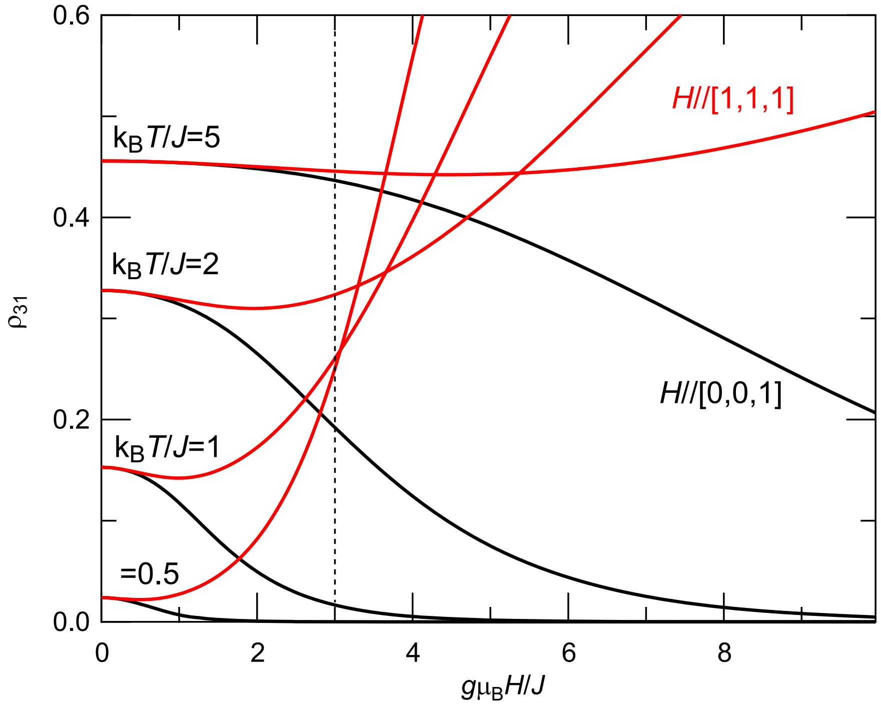


**Supplementary Figure 5.** Classical monopole density $\rho_{31}$ is plotted against normalized magnetic field $g\mu_B H/J$ for ***H***//[1,1,1] and [0,0,1]. Dotted vertical line indicates the field $g\mu_B H/J$=3, at which level crossing of 3-in-1-out and 2-in-2-out occurs when ***H*** is applied along [1,1,1].

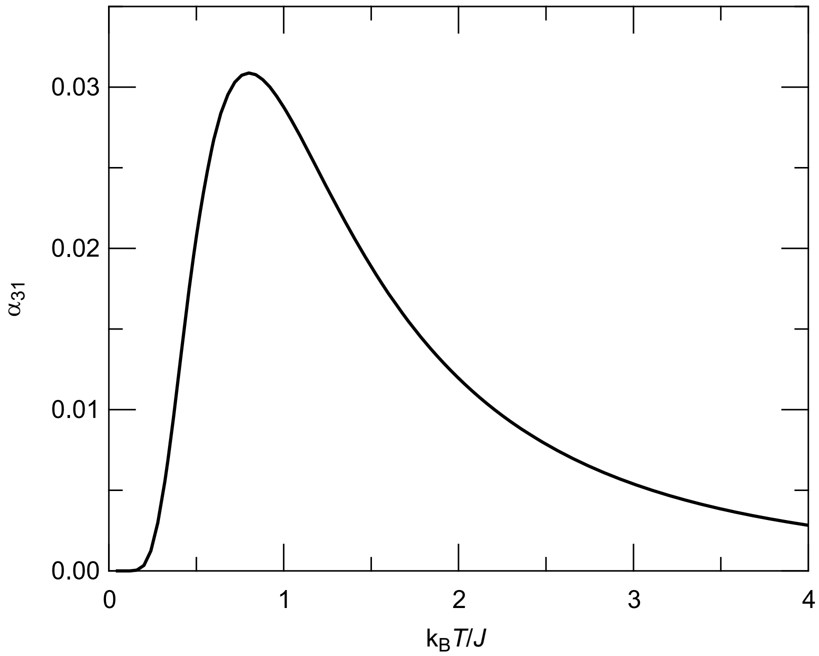


**Supplementary Figure 6.** ${h_J}^2$ coefficient of $\rho_{31}$, $\alpha_{31}$, is plotted against normalized

temperature, $k_B T/J$, where $h_J = g\mu_B H/J$.

| | h=0 | Energy | h ‖ [111] | Energy | h ‖ [001] | Energy |
|---|---|---|---|---|---|---|
| 2-in 2-out | 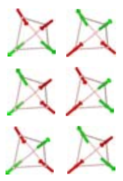 | -2J | 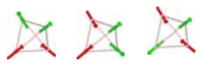 | -2J-4/3 h | 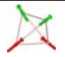 | -2J-4h/√3 |
| | | | | | 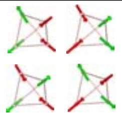 | -2J |
| | | | 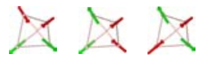 | -2J+4/3 h | | |
| | | | | | 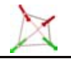 | -2J+4h/√3 |
| 3-in 1-out<br>1-in 3-out | 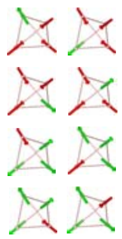 | 0 | 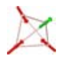 | -2h | 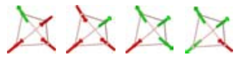 | -2h/√3 |
| | | | 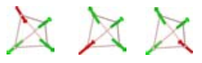 | -2/3 h | | |
| | | | 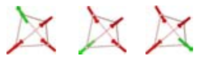 | 2/3 h | 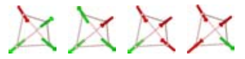 | 2h/√3 |
| | | | 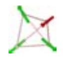 | 2h | | |
| All-in/All-out | 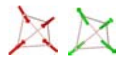 | 6J | 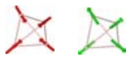 | 6J | 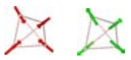 | 6J |

**Supplementary Table 1.** The energy of spin configurations in a single tetrahedron.

## Supplementary Note 1: Additional phonon scatterings in $Dy_2Ti_2O_7$

$\kappa/T$ of $Y_2Ti_2O_7$, $Dy_2Ti_2O_7$ at zero field and $Yb_2Ti_2O_7$ at 12 T are compared in Supplementary Figure 1. $\kappa/T$ of $Dy_2Ti_2O_7$ at high temperature is strongly suppressed from $Y_2Ti_2O_7$. Note that it decreases monotonically with $H$, indicating even smaller phonon $\kappa/T$ [1]. This implies the presence of unknown phonon scatterings, which are not likely caused by crystal field excitations because of the large energy gap of first excited state, ~380 K [2]. As discussed in the main text, $\kappa/T$ of $Yb_2Ti_2O_7$ at 12 T represents the purely phononic $\kappa/T$ at low temperatures, where $k_B T << g\mu_B H$. $\kappa/T$ at 2 K is much larger than $Dy_2Ti_2O_7$ and close to $Y_2Ti_2O_7$, suggesting the absence of such unknown scatterings.

## Supplementary Note 2: Field dependence of inelastic spin-phonon scattering rate

The leading process of inelastic spin-phonon scattering accompanies spin-flip due to $J_\perp$. This process involves incident and scattered monopoles with different energies and wave numbers within the monopole band, as depicted in Fig. 1**c** in the main text. This process requires flipping of spins. With increasing magnetic field, however, the alignment of spins along the field direction makes the spin flip difficult, due to the cost of increasing Zeeman energy. With the

reduction of this spin-flip scattering, the phonon thermal conductivity is expected to increase with the field, accordingly. The suppression of inelastic scattering in spin-ice systems is readily understood for $\boldsymbol{H}$//[0,0,1]. In this case, monopole density is expected to decrease monotonically (see Supplementary Figure 5). Such a suppression of scatterers causes an increase of phonon thermal conductivity. Therefore, the initial reduction of $\kappa(H)$ cannot be attributed to the inelastic spin-phonon scattering.

## Supplementary Note 3: Position of minimum in $\kappa(H)/T$

As shown in Figs. 3**c** and **d** in the main text, $\kappa(H)$ exhibits a minimum. The position of the minimum, $\mu_0 H_{min}$, is plotted against temperature in Supplementary Figure 2**a**. $\mu_0 H_{min}$ for $H$//[1,1,1] increases with temperature up to 0.5 K and saturates, whereas it increases monotonically for $H$//[0,0,1]. It should be noted that $\mu_0 H_{min}$ for $H$//[1,1,1] is reduced by a kink in $\kappa(H)/T$ at temperatures below 0.4 K due to the field-induced ferromagnetic ordering as discussed below with Supplementary Figure 3. Since the phonon thermal conductivity obeys the $H/T$ scaling as shown in Fig. 3**e** in the main text, it may be instructive to plot $\mu_0 H_{min}/T$ vs $T$. The observed strong variation of $\mu_0 H_{min}/T$ and the anisotropy are clearly inconsistent with the isotropic $H/T$ scaling. We point out that $\mu_0 H_{min}$ is determined by the competition between the monopole and phonon contributions, because the former decreases with the field, while the latter increases. Since the latter obeys the scaling, the inconsistency of $\mu_0 H_{min}(H)$ with the scaling is ascribed to the monopole contribution.

## Supplementary Note 4: Disappearance of initial reduction of $\kappa(H)/T$ with $H$ below $T_c$=0.19 K

In a temperature range of 0.2 K≤$T$≤0.3 K, a clear kink appears in $\kappa(H)/T$ for $\boldsymbol{H}$//[1,1,1] at the transition between spin liquid and ferromagnetic states, indicated by arrows in Supplementary Figure 3. The kink is shifted to lower field with decreasing temperature and vanishes below $T_c$ in the ferromagnetic state.

As discussed in the main text, the initial reduction of $\kappa(H)/T$ with $H$ observed above $T_c$ comes from the thermal conduction of magnetic quantum monopoles. The initial reduction in the temperature range 0.2 K≤$T$≤0.3 K is interrupted by the field-induced ferromagnetic ordering and $\kappa(H)/T$ shows a characteristic enhancement with field in the ferromagnetic state. The enhancement is understood by the suppression of elastic scattering of phonon due to the ordering of magnetic moments. As the kink disappears in the ferromagnetic state, the initial

reduction, which is the signature of monopole heat conduction, also disappears, in consistent with the suppression of spin-ice correlations below $T_c$ reported by the neutron scattering experiments [3]. The initial reduction disappears below $T_c$ also for ***H***//[0,0,1] (Supplementary Figure 4).

## Supplementary Note 5: Initial reduction of classical monopole density with magnetic field

By calculating the classical monopole density ($\rho_{31}$, 3-in-1-out and 1-in-3-out configurations) in magnetic field, we show that $\rho_{31}$ decreases with $H^2$ at zero-field limit, regardless of field direction.

Hamiltonian of a nearest-neighbor spin ice model is written as

$$H = J \sum_{<i,j>} \sigma_i^z \sigma_j^z - g\mu_B \boldsymbol{H} \cdot \sum_j \boldsymbol{S}_j$$

where $J$, which corresponds to $J_{\parallel}$ in the main text, is the nearest neighbor Ising interaction, the spin $\mathbf{S}_j$ is an Ising spin: $\boldsymbol{S}_j = \sigma_j^z \boldsymbol{d}_j$, $\sigma_j^z = \pm 1$, with anisotropy axes, $\boldsymbol{d}_0 = \frac{1}{\sqrt{3}}[1,1,1]$, $\boldsymbol{d}_1 = \frac{1}{\sqrt{3}}[1,-1,-1]$, $\boldsymbol{d}_2 = \frac{1}{\sqrt{3}}[-1,1,-1]$, $\boldsymbol{d}_3 = \frac{1}{\sqrt{3}}[-1,-1,1]$. With $h = g\mu_B H$, the energy of all the spin configurations are shown in Supplementary Table 1.

For ***H***//[1,1,1], $\rho_{31}$ is derived as,

$$\rho_{31} = \frac{8\cosh^3(2h_J/3t)}{N_{[1,1,1]}}$$

$$N_{[1,1,1]} = 6\exp\left(\frac{2}{t}\right)\cosh\left(\frac{4h_J}{3t}\right) + 8\cosh^3\left(\frac{2h_J}{3t}\right) + 2\exp\left(-\frac{6}{t}\right)$$

Here, $h_J = h/J$ and $t = k_B T/J$ are normalized field and temperature, respectively. For ***H***//[0,0,1],

$$\rho_{31} = \frac{8\cosh(2h_J/\sqrt{3}t)}{N_{[0,0,1]}}$$

$$N_{[0,0,1]} = 2\exp\left(\frac{2}{t}\right)\left[2 + \cosh\left(\frac{4h_J}{\sqrt{3}t}\right)\right] + 8\cosh\left(\frac{2h_J}{\sqrt{3}t}\right) + 2\exp\left(-\frac{6}{t}\right)$$

The resulting field dependences at different temperatures are plotted in Supplementary Figure 5. For ***H***//[0,0,1], the energy of one 2-in-2-out configuration decreases the most by Zeeman effect, leading to monotonic increase of 2-in-2-out density. As a result, $\rho_{31}$ decreases monotonically.

For $\boldsymbol{H}$//[1,1,1], the energy of one 3-in-1-out configuration decreases the most and crosses with the lowest energy of 2-in-2-out configuration at $g\mu_B H/J$=3. As this crossing occurs, $\rho_{31}$ rapidly increases with $H$. On the other hand, in low field region, $\rho_{31}$ decreases with field isotropically. We verify this by expanding $\rho_{31}$ with $h_J$ around $h_J = 0$. For both the two field directions, $h_J$-linear term vanishes and ${h_J}^2$ term is identical. The isotropic field dependence of $\rho_{31}$ at zero-field limit is then,

$$\rho_{31}(h_J) = \rho_{31}(0) - \alpha_{31}{h_J}^2 \dots$$

$$\alpha_{31} = \frac{8}{3t^2}\frac{\exp(2/\mathrm{t}) - \exp(-6/\mathrm{t})}{(\exp(-6/\mathrm{t}) + 3\exp(2/\mathrm{t}) + 4)^2}$$

The ${h_J}^2$ coefficient $\alpha_{31}$ is plotted against $t = k_B T/J$ in Supplementary Figure 6. It exhibits a maximum at $k_B T_{\max\alpha_{31}}/J = 0.8$. The relation between $T_{\max\alpha_{31}}$ and the monopole excitation energy $\Delta_{31}$ is then $\Delta_{31} = 2J = 2.5 k_B T_{\max\alpha_{31}}$. In the main text, the initial $H^2$ decrease of $\kappa/T$ is ascribed to decreasing number of monopoles and the $H^2$-coefficient $\alpha$ in the field dependence of $\kappa/T$ exhibits a maximum at $T_{\max}$ =0.3-0.5 K. If $T_{\max}$ is related to the monopole gap energy, it corresponds to 0.75-1.25 K of monopole excitation energy, which is strongly suppressed from the classical one, $2J_{\parallel}$=4 K [4]. It should be noted, however, that this estimation is of purely classical monopole.

## Supplementary References


[1] Kolland, G., Valldor, M., Hiertz, M., Frielingsdorf, J. & Lorenz, T. Anisotropic heat transport via monopoles in the spin-ice compound $Dy_2Ti_2O_7$. Phys. Rev. B **88**, 054406 (2013)

[2] Rosenkranz, S., Ramirez, A. P., Hayashi, A., Cava, R. J., Siddharthan, R. & Shastry B. S. Crystal-field interaction in the pyrochlore magnet $Ho_2Ti_2O_7$. J. Appl. Phys. **87**, 5914-5916 (2000)

[3] Chang, L.-J., Onoda, S., Su, Y., Kao, Y. -J., Tsuei, K. -D., Yasui, Y., Kakurai, K. & Lees, M. R. Higgs transition from a magnetic Coulomb liquid to a ferromagnet in $Yb_2Ti_2O_7$. Nat. Commun. **3**, 992 (2012).

[4] Ross, K. A., Savary, L., Gaulin, B. D. & Balents, L. Quantum excitations in quantum spin ice. Phys. Rev. X **1**, 021002 (2011).
